# Androgen-Targeted Therapy-Induced Epithelial Mesenchymal Plasticity and Neuroendocrine Transdifferentiation in Prostate Cancer: An Opportunity for Intervention

**DOI:** 10.3389/fonc.2014.00370

**Published:** 2014-12-23

**Authors:** Mannan Nouri, Ellca Ratther, Nataly Stylianou, Colleen C. Nelson, Brett G. Hollier, Elizabeth D. Williams

**Affiliations:** ^1^Vancouver Prostate Centre, Vancouver, BC, Canada; ^2^The University of British Columbia, Vancouver, BC, Canada; ^3^Australian Prostate Cancer Research Centre Queensland, Institute of Health and Biomedical Innovation, Princess Alexandra Hospital, Queensland University of Technology, Brisbane, QLD, Australia; ^4^Australian Prostate Cancer Research Centre Queensland, Translational Research Institute, Princess Alexandra Hospital, Queensland University of Technology, Brisbane, QLD, Australia; ^5^Department of Surgery, St Vincent’s Hospital, The University of Melbourne, Melbourne, VIC, Australia; ^6^Monash University, Melbourne, VIC, Australia

**Keywords:** prostate cancer, epithelial-to-mesenchymal transition, neuroendocrine, androgen deprivation therapy, castrate resistant, tumor cell plasticity, brachyury, Axl

## Abstract

Androgens regulate biological pathways to promote proliferation, differentiation, and survival of benign and malignant prostate tissue. Androgen receptor (AR) targeted therapies exploit this dependence and are used in advanced prostate cancer to control disease progression. Contemporary treatment regimens involve sequential use of inhibitors of androgen synthesis or AR function. Although targeting the androgen axis has clear therapeutic benefit, its effectiveness is temporary, as prostate tumor cells adapt to survive and grow. The removal of androgens (androgen deprivation) has been shown to activate both epithelial-to-mesenchymal transition (EMT) and neuroendocrine transdifferentiation (NEtD) programs. EMT has established roles in promoting biological phenotypes associated with tumor progression (migration/invasion, tumor cell survival, cancer stem cell-like properties, resistance to radiation and chemotherapy) in multiple human cancer types. NEtD in prostate cancer is associated with resistance to therapy, visceral metastasis, and aggressive disease. Thus, activation of these programs via inhibition of the androgen axis provides a mechanism by which tumor cells can adapt to promote disease recurrence and progression. Brachyury, Axl, MEK, and Aurora kinase A are molecular drivers of these programs, and inhibitors are currently in clinical trials to determine therapeutic applications. Understanding tumor cell plasticity will be important in further defining the rational use of androgen-targeted therapies clinically and provides an opportunity for intervention to prolong survival of men with metastatic prostate cancer.

## Introduction

Prostate cancer is the most prevalent malignancy in men, and ranks second as the cause of cancer-related deaths in the developed world (1, 2). Advanced prostate cancer is initially treated with androgen deprivation therapy (ADT) and subsequently with newer generation androgen-targeted therapies (ATT), approaches which rely on the central role of androgens in tumor development and growth. In the majority of patients, castrate resistant prostate cancer (CRPC) develops and tumor progression occurs despite treatment. The development of agents that more effectively block androgen receptor (AR) activity, such as enzalutamide and abiraterone, has greatly enhanced the clinical armamentarium and extended survival (3–6). Nonetheless, advanced prostate cancer remains incurable. Tumor cell plasticity induced by androgen deprivation may play a critical role in disease progression, and potentially provides an additional opportunity to further improve cancer control.

## Progression to Castrate Resistance

**Figure 1:**
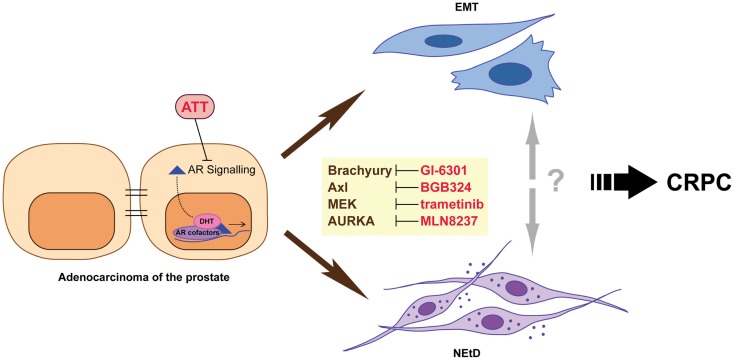
**Inhibition of androgen receptor (AR) signaling using androgen-targeted therapies (ATT) induces adaptive responses including epithelial–mesenchymal transition (EMT) and neuroendocrine transdifferentiation (NEtD) in prostate cancer cells**. These phenotypes are associated with CRPC (castrate resistance prostate cancer). Inhibition of plasticity drivers Brachyury, Axl, MEK, and Aurora kinase A provide potential mechanisms to reduce the induction of the EMT and/or NEtD phenotypes.

While the exact mechanisms underlying the development of CRPC are not yet known, it arises when cancer cells can either maintain AR signaling in the absence of normal levels of ligand or no longer require activation of this pathway for survival and proliferation. There are a number of mechanisms that can produce this outcome, including altered functionality of the AR due to genomic events, resulting in either promiscuous (7, 8), constitutively activated (9, 10), or hypersensitive (11, 12) states; intraprostatic production of androgens by tumor cells themselves (13); and altered growth factor and/or microenvironment signaling (14–18). Despite the development of multiple strategies that effectively target the androgen axis, disease progression is inevitable. This is underpinned by the accumulation of further genomic abnormalities, outgrowth of different clonal populations of tumor cells, and the adaptive response of cancer cells to therapy. In this review, we focus on adaptive changes induced by therapy, specifically epithelial-to-mesenchymal plasticity (EMP) and neuroendocrine transdifferentiation (NEtD), which may contribute to the development of advanced disease (Figure 1). A better understanding of these processes will contribute to the development of new therapeutic strategies that may potentially enhance the efficacy of androgen-targeted agents and delay disease progression.

## Epithelial-To-Mesenchymal Plasticity

Epithelial-to-mesenchymal transition (EMT) is a process by which adherent, polar cells with an otherwise epithelial phenotype develop more migratory and invasive properties through altered gene expression (19–23). Both EMT and the related process mesenchymal-to-epithelial transition are physiological mechanisms important in development and tissue repair. However, when differentiated epithelium begins to display mesenchymal characteristics it is often a sign of disease progression in cancers (19, 24–27). EMT is commonly characterized by the loss of epithelial markers (typically E-cadherin, epithelial cytokeratins, and desmosomes), and gain of mesenchymal markers (such as N-cadherin, vimentin, and fibronectin) and transcriptional repressors of E-cadherin (Twist1, Snai1, Snai2, Zeb1, Zeb2) (20, 21). EMT has been associated with advanced prostate cancer, and correlated with aggressive behavior and therapy resistance in primary tumors (17, 28–30).

## Neuroendocrine Transdifferentiation

While men may present with prostate cancer demonstrating various neuroendocrine features (31), the prevalence of neuroendocrine differentiation increases following ADT and in CRPC (32–37). These cells not only express neuropeptides, reminiscent of the normal NE cells of the prostate, but also proteins that are characteristic of prostate epithelial cells [such as prostatic acid phosphatase cytokeratin 8/18 and/or epithelial adhesion molecules and proliferation markers (38, 39)], while AR expression is typically absent or low (40). Importantly, the number of NE-like prostate cancer cells is positively associated with the duration of hormone deprivation therapy (32–34). There are several hypotheses for the origin of NE-like prostate cancer cells. It has been postulated that NE-like cancer cells can arise during disease progression from NE cells of the prostate (41). However, the observation that genetic aberrations are common to both the adenocarcinoma and NE-like cells (42–45) suggests that this is not likely to be a common mechanism. An alternative explanation is that a common progenitor prostate cancer stem cell gives rise to both the NE-like and adenocarcinoma components and both these components continue to evolve and respond to selective pressures in parallel (42, 44, 46, 47). In contrast, NEtD is a process that can enable prostatic adenocarcinoma cells to gain NE characteristics without relying on genetic divergence. NEtD can occur after prolonged androgen deprivation, and has recently been reported in a patient derived xenograft (48). This mechanism would potentially enable tumor cells to reduce ATT-induced apoptosis and thus provide an adaptive pathway that would contribute to the development of CRPC (41).

## Androgens Suppress Neuroendocrine Transdifferentiation

Evidence of NEtD has been observed in both *in vitro* and *in vivo* studies. LNCaP cells, an androgen dependent prostate cancer cell line derived from a lymph node metastasis, undergo NEtD when exposed to media lacking androgens (39, 49–51). In low-androgen conditions, LNCaP cells take on an altered elongated neuron-like phenotype, gain cytoplasmic secretory granules, and undergo growth arrest. This is accompanied by an increase in expression of NE markers and a decrease in AR and PSA levels. This transdifferentiation is reversible with the addition of androgens (DHT) to the media, an observation consistent with the identical allelic profiles of NEtD LNCaP and parental LNCaP cells. Silencing of the AR using siRNA also induces NEtD in LNCaP cells, suggesting that AR signaling suppresses NEtD (52). *In vivo* studies also provide support for the NEtD model. Castration of nude mice bearing prostate cancer xenografts LNCaP, PC-295, CWR22, and PC-310 increased the number of tumor cells expressing NE markers, consistent with induction of NEtD (53–56). Furthermore, implantation of primary patient tumor tissues from a population of adenocarcinoma cells implanted under the renal capsule of castrated mice appear to undergo an NEtD en masse as an adaptive response (48).

## Therapy-Induced EMT

There is accumulating evidence supporting that ADT may induce an EMT, and that this is particularly prominent with the newer generation ATT. ADT has been associated with an increase in the expression of mesenchymal markers N-cadherin, vimentin, Zeb1, Twist1, and Snai2, with a concomitant loss of E-cadherin in patient derived xenografts and clinical prostate tumors (17, 57–59). Traditionally, investigations have primarily focused on the effects of targeting AR signaling in prostate cancer cells; however, ADT/ATT is not specific to tumor cells alone. Recent reports demonstrate significant effects of ADT/ATT on the tumor microenvironment, including stromal and immune cells (15, 18, 60). For instance, elevated numbers of tumor associated macrophages have been reported in men undergoing ADT (60), and these cells have been shown to promote local invasion and metastatic dissemination of tumor cells in response to ADT (18, 57–60). Hence, the implications of targeting the androgen axis and its effect on the multiple cell types comprising the tumor microenvironment needs to be assessed when considering therapeutic interventions.

## Therapy-Induced EMT and Neuroendocrine Transdifferentiation as Clinical Targets

Therapeutically targeting regulators of EMP/NEtD is an attractive concept that has recently matured to clinical trials (Figure 1). Brachyury is a transcription factor required for the developmental EMT that generates mesoderm by converting epithelial cells into migratory mesenchymal cells (61). In tumor cells, including prostate cancer, Brachyury also induces EMT and an invasive phenotype (62–65). Furthermore, Brachyury is overexpressed at both the transcript and protein level in clinical prostate cancer specimens, and nuclear expression is associated with metastasis (66). While the regulation of Brachyury by androgen-targeted therapies has not been addressed, Brachyury motifs were highly enriched in AR bound promoters when LAPC-4 cells were grown in the presence of AR antagonist flutamide (67). Furthermore, *in silico* bioinformatic analysis using transcriptional profiles from clinical prostate cancer specimens and clustering Brachyury co-expressed genes by functional role/signaling pathways demonstrated an enrichment for regulation of neuron differentiation and nervous system development (68). An inverse relationship between Brachyury and E-cadherin expression, with a concomitant positive correlation of Brachyury with EMT promoting genes FN1, Snai1, IL8, and TGF-β was also observed. Thus, we hypothesize that targeting Brachyury in the context of ATT may modulate the emergence of both a neuroendocrine phenotype and EMP by preventing, for example, the induction of Brachyury mediated release of migration/invasion promoting soluble factors into the tumor microenvironment (62, 68, 69). GI-6301 (Tarmogen) is a Brachyury vaccine (70) currently in Phase I clinical trial in patients with metastatic or unresectable locally recurrent cancers who have failed previous therapy or have no further therapeutic options (NCT01519817). Recent assessment of data from patients with advanced chordoma in this trial demonstrated safety and a confirmed partial response (71), and data from the larger cohort are eagerly awaited.

The receptor tyrosine kinase Axl is implicated in the Snai1-, Snai2-, IL6-, and STAT3-mediated activation of EMT (72, 73) as well as the metastasis promoting AKT/NF-κB and AKT/Snai2 pathways (73, 74) in multiple cancer types. Targeting Axl has shown promise in preclinical models of cancer progression (75–77), and clinical trials are currently underway. BGB324 is a small molecule inhibitor of the Axl receptor tyrosine kinase developed to block EMT with the goal of inhibiting drug-resistance and metastasis. Recent Phase Ia data have demonstrated BGB324 to be safe and well tolerated, and Phase Ib studies commenced in non-small cell lung cancer and acute myeloid leukemia in 2014. Cabozantinib is another tyrosine kinase inhibitor targeting Axl, as well as EMT promoting kinases VEGFR2, RET, KIT, FLT-1/3/4, c-MET, and Tie-2 (78–80). Clinically significant regression of metastatic tumors in CRPC patients was achieved with cabozantinib treatment in a Phase II trial (81). Of course the precise molecular mechanism underpinning this efficacy is not clear and likely involves inhibition of multiple tyrosine kinases in several cell types. Trials investigating whether cabozantinib is a useful addition to ADT in the control of prostate cancer are currently underway (NCT01630590).

MEK inhibitors may also be useful in managing therapy-induced EMP/NEtD. *In vitro*, MEK inhibitor PD98059 blocked the acquisition of NE-like morphology and prevented the increase in NSE levels usually observed in LNCaP-C33 cells induced to undergo NEtD by androgen-depletion (82). Ectopic expression of constitutively active AR in LNCaP cells inhibited RAF/MEK/ERK-induced NSE expression (83), demonstrating the central regulatory role of AR in constraining the emergence of this phenotype. Furthermore, the RAF/MEK/ERK pathway has been shown to be necessary for the induction of Twist1, Snai1, and N-cadherin in multiple cancer models (84, 85). A neoadjuvant trial examining the effect of short-term MEK inhibition (trametinib) prior to radical prostatectomy in the context of ADT on markers of EMT (N-cadherin, vimentin) has recently commenced (NCT01990196).

Finally, Aurora kinase A (AURKA) inhibitors may also be effective in inhibiting ATT-induced EMP/NEtD as they suppress both EMT and NEtD *in vitro* and *in vivo* (86, 87). In cancer cells, AURKA has been demonstrated to play an important role in the genesis of a more mesenchymal phenotype via down-regulation of E-cadherin and up-regulation of vimentin (88). Clinical trials examining the role of the inhibitors in prostate cancer are currently ongoing (NCT01799278, NCT01094288).

Despite independent lines of evidence implicating key factors in both EMT and NEtD, the functional and molecular relationship between these states in prostate cancer has not been extensively explored. McKeithen et al. (89) have demonstrated that the well-established EMT-inducing transcription factor Snai1 induced both EMT and NEtD in LNCaP cells as defined by morphology and marker expression. However, as the data are mostly presented as analyses of bulk populations of cells, it is not possible to determine whether EMT and NEtD phenotypes are co-expressed within individual cells, and are thus intimately linked, or whether these transdifferentiation processes are independent of each other and become activated by influences such as neighboring cells, local microenvironmental cues, or cell intrinsic factors.

## Concluding Remarks

Multiple factors are clearly involved in the progression to CRPC during treatment with ATT. Studies over the past two decades have associated blockade of the androgen axis with the increased prevalence of neuroendocrine prostate cancer. These observations, in combination with recent reports of androgen deprivation modulating EMP, suggest novel strategies for therapeutic intervention. Further studies will be required to determine whether these adaptive response pathways have a functional role in the progression to CRPC or are simply a consequence of removing the differentiation pressure imposed by active androgen signaling on prostate cells. Moreover, revealing if and how these plasticity pathways intersect in the androgen-targeted environment will be an intriguing area for future research. Improved understanding of the molecular pathways underlying the adaptive responses to ATT provides opportunities to investigate whether targeted inhibition of these pathways will delay tumor progression and thus improve outcomes for men with prostate cancer.

## Conflict of Interest Statement

The authors declare that the research was conducted in the absence of any commercial or financial relationships that could be construed as a potential conflict of interest.

## References

[1] Jemal A Siegel R Xu J Ward E Cancer statistics, 2010. CA Cancer J Clin (2010) 60:277–300 10.3322/caac.20073 20610543

[2] Siegel R Ma J Zou Z Jemal A Cancer statistics, 2014. CA Cancer J Clin (2014) 64:9–29 10.3322/caac.21208 24399786

[3] Dreicer R MacLean D Suri A Stadler WM Shevrin D Hart L Phase I/II trial of orteronel (TAK-700) – an investigational 17,20-lyase inhibitor – in patients with metastatic castration-resistant prostate cancer. Clin Cancer Res (2014) 20:1335–44. 10.1158/1078-0432.CCR-13-2436 24418642

[4] Joseph JD Lu N Qian J Sensintaffar J Shao G Brigham D A clinically relevant androgen receptor mutation confers resistance to second-generation antiandrogens enzalutamide and ARN-509. Cancer Discov (2013) 3:1020–9. 10.1158/2159-8290.CD-13-0226 23779130

[5] Rathkopf DE Morris MJ Fox JJ Danila DC Slovin SF Hager JH Phase I study of ARN-509, a novel antiandrogen, in the treatment of castration-resistant prostate cancer. J Clin Oncol (2013) 31:3525–30. 10.1200/JCO.2013.50.1684 24002508 PMC3782148

[6] Heidenreich A Bastian PJ Bellmunt J Bolla M Joniau S van der Kwast T EAU guidelines on prostate cancer. Part II: treatment of advanced, relapsing, and castration-resistant prostate cancer. Eur Urol (2014) 65:467–79 10.1016/j.eururo.2013.11.002 24321502

[7] Fujimoto N Miyamoto H Mizokami A Harada S Nomura M Ueta Y Prostate cancer cells increase androgen sensitivity by increase in nuclear androgen receptor and androgen receptor coactivators; a possible mechanism of hormone-resistance of prostate cancer cells. Cancer Invest (2007) 25:32–7. 10.1080/07357900601130698 17364555

[8] Tsao CK Galsky MD Small AC Yee T Oh WK. Targeting the androgen receptor signalling axis in castration-resistant prostate cancer (CRPC). BJU Int (2012) 110:1580–8. 10.1111/j.1464-410X.2012.11445.x 22985411

[9] Attar RM Takimoto CH Gottardis MM. Castration-resistant prostate cancer: locking up the molecular escape routes. Clin Cancer Res (2009) 15:3251–5. 10.1158/1078-0432.CCR-08-1171 19447877

[10] Dehm SM Schmidt LJ Heemers HV Vessella RL Tindall DJ. Splicing of a novel androgen receptor exon generates a constitutively active androgen receptor that mediates prostate cancer therapy resistance. Cancer Res (2008) 68:5469–77. 10.1158/0008-5472.CAN-08-0594 18593950 PMC2663383

[11] Visakorpi T Hyytinen E Koivisto P Tanner M Keinanen R Palmberg C In vivo amplification of the androgen receptor gene and progression of human prostate cancer. Nat Genet (1995) 9:401–6 10.1038/ng0495-401 7795646

[12] Waltering KK Helenius MA Sahu B Manni V Linja MJ Janne OA Increased expression of androgen receptor sensitizes prostate cancer cells to low levels of androgens. Cancer Res (2009) 69:8141–9. 10.1158/0008-5472.CAN-09-0919 19808968

[13] Locke JA Guns ES Lubik AA Adomat HH Hendy SC Wood CA Androgen levels increase by intratumoral de novo steroidogenesis during progression of castration-resistant prostate cancer. Cancer Res (2008) 68:6407–15 10.1158/0008-5472.CAN-07-5997 18676866

[14] Lai JJ Lai KP Chuang KH Chang P Yu IC Lin WJ Monocyte/macrophage androgen receptor suppresses cutaneous wound healing in mice by enhancing local TNF-alpha expression. J Clin Invest (2009) 119:3739–51. 10.1172/JCI39335 19907077 PMC2786793

[15] Loberg RD Ying C Craig M Yan L Snyder LA Pienta KJ. CCL2 as an important mediator of prostate cancer growth in vivo through the regulation of macrophage infiltration. Neoplasia (2007) 9:556–62. 10.1593/neo.07307 17710158 PMC1939930

[16] Lubik AA Gunter JH Hollier BG Ettinger S Fazli L Stylianou N IGF2 increases de novo steroidogenesis in prostate cancer cells. Endocr Relat Cancer (2013) 20:173–86. 10.1530/ERC-12-0250 23319492

[17] Sun Y Wang BE Leong KG Yue P Li L Jhunjhunwala S Androgen deprivation causes epithelial-mesenchymal transition in the prostate: implications for androgen-deprivation therapy. Cancer Res (2012) 72:527–36. 10.1158/0008-5472.CAN-11-3004 22108827

[18] Zhu P Baek SH Bourk EM Ohgi KA Garcia-Bassets I Sanjo H Macrophage/cancer cell interactions mediate hormone resistance by a nuclear receptor derepression pathway. Cell (2006) 124:615–29. 10.1016/j.cell.2005.12.032 16469706

[19] Aktas B Tewes M Fehm T Hauch S Kimmig R Kasimir-Bauer S. Stem cell and epithelial-mesenchymal transition markers are frequently overexpressed in circulating tumor cells of metastatic breast cancer patients. Breast Cancer Res (2009) 11:R46. 10.1186/bcr2333 19589136 PMC2750105

[20] Chaffer CL Thompson EW Williams ED Mesenchymal to epithelial transition in development and disease. Cells Tissues Organs (2007) 185:7–19 10.1159/000101298 17587803

[21] Hugo H Ackland ML Blick T Lawrence MG Clements JA Williams ED Epithelial-mesenchymal and mesenchymal-epithelial transitions in carcinoma progression. J Cell Physiol (2007) 213:374–83. 10.1002/jcp.21223 17680632

[22] Said NABM Williams ED Remodelling the malignant phenotype: impact of EMT. Drug Discov Today Dis Models (2009) 6:21–5 10.1016/j.ddmod.2008.12.002

[23] Thompson EW Williams ED. EMT and MET in carcinoma-clinical observations, regulatory pathways and new models. Clin Exp Metastasis (2008) 25:591–2. 10.1007/s10585-008-9189-8 18566898

[24] Bhangu A Wood G Mirnezami A Darzi A Tekkis P Goldin R. Epithelial mesenchymal transition in colorectal cancer: seminal role in promoting disease progression and resistance to neoadjuvant therapy. Surg Oncol (2012) 21:316–23. 10.1016/j.suronc.2012.08.003 22981546

[25] Ding W Nowakowski GS Knox TR Boysen JC Maas ML Schwager SM Bi-directional activation between mesenchymal stem cells and CLL B-cells: implication for CLL disease progression. Br J Haematol (2009) 147:471–83. 10.1111/j.1365-2141.2009.07868.x 19751240 PMC2783570

[26] Kim SH Yu MA Ryu ES Jang YH Kang DH. Indoxyl sulfate-induced epithelial-to-mesenchymal transition and apoptosis of renal tubular cells as novel mechanisms of progression of renal disease. Lab Invest (2012) 92:488–98. 10.1038/labinvest.2011.194 22231736

[27] Turley EA Veiseh M Radisky DC Bissell MJ. Mechanisms of disease: epithelial-mesenchymal transition-does cellular plasticity fuel neoplastic progression? Nat Clin Pract Oncol (2008) 5:280–90. 10.1038/ncponc1089 18349857 PMC2846172

[28] Das R Gregory PA Hollier BG Tilley WD Selth LA. Epithelial plasticity in prostate cancer: principles and clinical perspectives. Trends Mol Med (2014) 20(11):643–51. 10.1016/j.molmed.2014.09.004 25262538

[29] Marin-Aguilera M Codony-Servat J Reig O Lozano JJ Fernandez PL Pereira MV Epithelial-to-mesenchymal transition mediates docetaxel resistance and high risk of relapse in prostate cancer. Mol Cancer Ther (2014) 13:1270–84. 10.1158/1535-7163.MCT-13-0775 24659820

[30] Ren D Wang M Guo W Huang S Wang Z Zhao X Double-negative feedback loop between ZEB2 and miR-145 regulates epithelial-mesenchymal transition and stem cell properties in prostate cancer cells. Cell Tissue Res (2014) 358(3):763–78. 10.1007/s00441-014-2001-y 25296715

[31] Epstein JI Amin MB Beltran H Lotan TL Mosquera JM Reuter VE Proposed morphologic classification of prostate cancer with neuroendocrine differentiation. Am J Surg Pathol (2014) 38:756–67. 10.1097/PAS.0000000000000208 24705311 PMC4112087

[32] Abrahamsson PA Falkmer S Falt K Grimelius L. The course of neuroendocrine differentiation in prostatic carcinomas. An immunohistochemical study testing chromogranin A as an “endocrine marker”. Pathol Res Pract (1989) 185:373–80. 10.1016/s0344-0338(89)80016-0 2813190

[33] Hirano D Okada Y Minei S Takimoto Y Nemoto N. Neuroendocrine differentiation in hormone refractory prostate cancer following androgen deprivation therapy. Eur Urol (2004) 45:586–92. 10.1016/j.eururo.2003.11.032 15082200

[34] Ito T Yamamoto S Ohno Y Namiki K Aizawa T Akiyama A Up-regulation of neuroendocrine differentiation in prostate cancer after androgen deprivation therapy, degree and androgen independence. Oncol Rep (2001) 8:1221. 11605036 10.3892/or.8.6.1221

[35] Jiborn T Bjartell A Abrahamsson PA. Neuroendocrine differentiation in prostatic carcinoma during hormonal treatment. Urology (1998) 51:585–9. 10.1016/S0090-4295(97)00684-5 9586611

[36] di Sant’Agnese PA. Neuroendocrine differentiation in carcinoma of the prostate. Diagnostic, prognostic, and therapeutic implications. Cancer (1992) 70:254–68. 10.1002/1097-0142(19920701)70:1+<254::AID-CNCR2820701312>3.0.CO;2-E 1350941

[37] Wafa LA Palmer J Fazli L Hurtado-Coll A Bell RH Nelson CC Comprehensive expression analysis of L-DOPA decarboxylase and established neuroendocrine markers in neoadjuvant hormone-treated versus varying Gleason grade prostate tumors. Hum Pathol (2007) 38:161–70. 10.1016/j.humpath.2006.07.003 16997353

[38] Vashchenko N Abrahamsson PA. Neuroendocrine differentiation in prostate cancer: implications for new treatment modalities. Eur Urol (2005) 47:147–55. 10.1016/j.eururo.2004.09.007 15661408

[39] Yuan T-C Veeramani S Lin F-F Kondrikou D Zelivianski S Igawa T Androgen deprivation induces human prostate epithelial neuroendocrine differentiation of androgen-sensitive LNCaP cells. Endocr Relat Cancer (2006) 13:151–67. 10.1677/erc.1.01043 16601285

[40] Huang J Yao JL di Sant’Agnese PA Yang Q Bourne PA Na Y. Immunohistochemical characterization of neuroendocrine cells in prostate cancer. Prostate (2006) 66:1399–406. 10.1002/pros.20434 16865726

[41] Beltran H Tomlins S Aparicio A Arora V Rickman D Ayala G Aggressive variants of castration-resistant prostate cancer. Clin Cancer Res (2014) 20:2846–50. 10.1158/1078-0432.ccr-13-3309 24727321 PMC4040316

[42] Hansel DE Nakayama M Luo J Abukhdeir AM Park BH Bieberich CJ Shared TP53 gene mutation in morphologically and phenotypically distinct concurrent primary small cell neuroendocrine carcinoma and adenocarcinoma of the prostate. Prostate (2009) 69:603–9. 10.1002/pros.20910 19125417 PMC3170854

[43] Lotan TL Gupta NS Wang W Toubaji A Haffner MC Chaux A ERG gene rearrangements are common in prostatic small cell carcinomas. Mod Pathol (2011) 24:820–8. 10.1038/modpathol.2011.7 21336263 PMC3484363

[44] Palmgren JS Karavadia SS Wakefield MR. Unusual and underappreciated: small cell carcinoma of the prostate. Semin Oncol (2007) 34:22–9. 10.1053/j.seminoncol.2006.10.026 17270662

[45] Williamson SR Zhang S Yao JL Huang J Lopez-Beltran A Shen S ERG-TMPRSS2 rearrangement is shared by concurrent prostatic adenocarcinoma and prostatic small cell carcinoma and absent in small cell carcinoma of the urinary bladder: evidence supporting monoclonal origin. Mod Pathol (2011) 24:1120–7. 10.1038/modpathol.2011.56 21499238 PMC3441178

[46] Bonkhoff H Remberger K. Differentiation pathways and histogenetic aspects of normal and abnormal prostatic growth: a stem cell model. Prostate (1996) 28:98–106. 10.1002/(SICI)1097-0045(199602)28:2<98:AID-PROS4>3.0.CO;2-J 8604398

[47] Bonkhoff H Wernert N Dhom G Remberger K. Relation of endocrine-paracrine cells to cell proliferation in normal, hyperplastic, and neoplastic human prostate. Prostate (1991) 19:91–8. 10.1002/pros.2990190202 1717965

[48] Lin D Wyatt AW Xue H Wang Y Dong X Haegert A High fidelity patient-derived xenografts for accelerating prostate cancer discovery and drug development. Cancer Res (2014) 74:1272–83. 10.1158/0008-5472.CAN-13-2921-T 24356420

[49] Horoszewicz JS Leong SS Kawinski E Karr JP Rosenthal H Chu TM LNCaP model of human prostatic carcinoma. Cancer Res (1983) 43:1809–18. 6831420

[50] Shen R Dorai T Szaboles M Katz AE Olsson CA Buttyan R. Transdifferentiation of cultured human prostate cancer cells to a neuroendocrine cell phenotype in a hormone-depleted medium. Urol Oncol (1997) 3:67–75. 10.1016/S1078-1439(97)00039-2 21227062

[51] Zelivianski S Verni M Moore C Kondrikov D Taylor R Lin M-F. Multipathways for transdifferentiation of human prostate cancer cells into neuroendocrine-like phenotype. Biochim Biophys Acta (2001) 1539:28–43. 10.1016/S0167-4889(01)00087-8 11389966

[52] Wright ME Tsai M-J Aebersold R. Androgen receptor represses the neuroendocrine transdifferentiation process in prostate cancer cells. Mol Endocrinol (2003) 17:1726–37. 10.1210/me.2003-0031 12775765

[53] Burchardt T Burchardt M Chen MW Cao Y de la Taille A Shabsigh A Transdifferentiation of prostate cancer cells to a neuroendocrine cell phenotype in vitro and in vivo. J Urol (1999) 162:1800–5. 10.1016/S0022-5347(05)68241-9 10524938

[54] Jongsma J Oomen MH Noordzij MA Van Weerden WM Martens GJM van der Kwast TH Kinetics of neuroendocrine differentiation in an androgen-dependent human prostate xenograft model. Am J Pathol (1999) 154:543–51. 10.1016/S0002-9440(10)65300-X 10027412 PMC1850014

[55] Huss WJ Gregory CW Smith GJ. Neuroendocrine cell differentiation in the CWR22 human prostate cancer xenograft: association with tumor cell proliferation prior to recurrence. Prostate (2004) 60:91–7. 10.1002/pros.20032 15162375

[56] Jongsma J Oomen MH Noordzij MA Van Weerden WM Martens GJ van der Kwast TH Androgen deprivation of the PC-310 human prostate cancer model system induces neuroendocrine differentiation. Cancer Res (2000) 60:741–8. 10676662

[57] Izumi K Fang LY Mizokami A Namiki M Li L Lin WJ Targeting the androgen receptor with siRNA promotes prostate cancer metastasis through enhanced macrophage recruitment via CCL2/CCR2-induced STAT3 activation. EMBO Mol Med (2013) 5:1383–401. 10.1002/emmm.201202367 23982944 PMC3799493

[58] Lin TH Izumi K Lee SO Lin WJ Yeh S Chang C. Anti-androgen receptor ASC-J9 versus anti-androgens MDV3100 (enzalutamide) or casodex (bicalutamide) leads to opposite effects on prostate cancer metastasis via differential modulation of macrophage infiltration and STAT3-CCL2 signaling. Cell Death Dis (2013) 4:e764. 10.1038/cddis.2013.270 23928703 PMC3763432

[59] Lin TH Lee SO Niu Y Xu D Liang L Li L Differential androgen deprivation therapies with anti-androgens casodex/bicalutamide or MDV3100/enzalutamide versus anti-androgen receptor ASC-J9(R) lead to promotion versus suppression of prostate cancer metastasis. J Biol Chem (2013) 288:19359–69. 10.1074/jbc.M113.477216 23687298 PMC3707641

[60] Gannon PO Poisson AO Delvoye N Lapointe R Mes-Masson AM Saad F. Characterization of the intra-prostatic immune cell infiltration in androgen-deprived prostate cancer patients. J Immunol Methods (2009) 348:9–17. 10.1016/j.jim.2009.06.004 19552894

[61] Yamada T. Caudalization by the amphibian organizer: Brachyury, convergent extension and retinoic acid. Development (1994) 120:3051–62. 7720551 10.1242/dev.120.11.3051

[62] Fernando RI Litzinger M Trono P Hamilton DH Schlom J Palena C. The T-box transcription factor Brachyury promotes epithelial-mesenchymal transition in human tumor cells. J Clin Invest (2010) 120:533–44. 10.1172/JCI38379 20071775 PMC2810072

[63] Imajyo I Sugiura T Kobayashi Y Shimoda M Ishii K Akimoto N T-box transcription factor Brachyury expression is correlated with epithelial-mesenchymal transition and lymph node metastasis in oral squamous cell carcinoma. Int J Oncol (2012) 41:1985–95. 10.3892/ijo.2012.1673 23076115 PMC3583627

[64] Roselli M Fernando RI Guadagni F Spila A Alessandroni J Palmirotta R Brachyury, a driver of the epithelial-mesenchymal transition, is overexpressed in human lung tumors: an opportunity for novel interventions against lung cancer. Clin Cancer Res (2012) 18:3868–79. 10.1158/1078-0432.CCR-11-3211 22611028 PMC3472640

[65] Shimoda M Sugiura T Imajyo I Ishii K Chigita S Seki K The T-box transcription factor Brachyury regulates epithelial-mesenchymal transition in association with cancer stem-like cells in adenoid cystic carcinoma cells. BMC Cancer (2012) 12:377. 10.1186/1471-2407-12-377 22931165 PMC3492149

[66] Thoma C Prostate cancer: Brachyury – a biomarker for progression and prognosis? Nat Rev Urol (2014) 11:485 10.1038/nrurol.2014.184 25069730

[67] Perets R Kaplan T Stein I Hidas G Tayeb S Avraham E Genome-wide analysis of androgen receptor targets reveals COUP-TF1 as a novel player in human prostate cancer. PLoS One (2012) 7:e46467. 10.1371/journal.pone.0046467 23056316 PMC3464259

[68] Pinto F Pertega-Gomes N Pereira MS Vizcaino JR Monteiro P Henrique RM T-box transcription factor Brachyury is associated with prostate cancer progression and aggressiveness. Clin Cancer Res (2014) 20:4949–61. 10.1158/1078-0432.CCR-14-0421 25009296

[69] Fernando RI Castillo MD Litzinger M Hamilton DH Palena C. IL-8 signaling plays a critical role in the epithelial-mesenchymal transition of human carcinoma cells. Cancer Res (2011) 71:5296–306. 10.1158/0008-5472.CAN-11-0156 21653678 PMC3148346

[70] Hamilton DH Litzinger MT Jales A Huang B Fernando RI Hodge JW Immunological targeting of tumor cells undergoing an epithelial-mesenchymal transition via a recombinant Brachyury-yeast vaccine. Oncotarget (2013) 4:1777–90. 24125763 10.18632/oncotarget.1295 PMC3858563

[71] Heery CR Singh H Jennifer L Marte JL Madan RA O’Sullivan Coyne GH NCI experience using yeast-Brachyury vaccine (GI-6301) in patients with advanced chordoma. J Clin Oncol (2014) 32:abstract 3081.

[72] Asiedu MK Beauchamp-Perez FD Ingle JN Behrens MD Radisky DC Knutson KL. Axl induces epithelial-to-mesenchymal transition and regulates the function of breast cancer stem cells. Oncogene (2014) 33:1316–24. 10.1038/onc.2013.57 23474758 PMC3994701

[73] Paccez JD Vasques GJ Correa RG Vasconcellos JF Duncan K Gu X The receptor tyrosine kinase Axl is an essential regulator of prostate cancer proliferation and tumor growth and represents a new therapeutic target. Oncogene (2013) 32:689–98. 10.1038/onc.2012.89 22410775 PMC4078100

[74] Li Y Jia L Ren D Liu C Gong Y Wang N Axl mediates tumor invasion and chemosensitivity through PI3K/Akt signaling pathway and is transcriptionally regulated by slug in breast carcinoma. IUBMB Life (2014) 66:507–18. 10.1002/iub.1285 24984960

[75] Holland SJ Pan A Franci C Hu Y Chang B Li W R428, a selective small molecule inhibitor of Axl kinase, blocks tumor spread and prolongs survival in models of metastatic breast cancer. Cancer Res (2010) 70:1544–54. 10.1158/0008-5472.CAN-09-2997 20145120

[76] Ye X Li Y Stawicki S Couto S Eastham-Anderson J Kallop D An anti-Axl monoclonal antibody attenuates xenograft tumor growth and enhances the effect of multiple anticancer therapies. Oncogene (2010) 29:5254–64. 10.1038/onc.2010.268 20603615

[77] Zhang YX Knyazev PG Cheburkin YV Sharma K Knyazev YP Orfi L AXL is a potential target for therapeutic intervention in breast cancer progression. Cancer Res (2008) 68:1905–15. 10.1158/0008-5472.CAN-07-2661 18339872

[78] Castellone MD Carlomagno F Salvatore G Santoro M. Receptor tyrosine kinase inhibitors in thyroid cancer. Best Pract Res Clin Endocrinol Metab (2008) 22:1023–38. 10.1016/j.beem.2008.09.012 19041829

[79] Timar J Dome B. Antiangiogenic drugs and tyrosine kinases. Anticancer Agents Med Chem (2008) 8:462–9. 10.2174/187152008784533035 18537529

[80] Yakes FM Chen J Tan J Yamaguchi K Shi Y Yu P Cabozantinib (XL184), a novel MET and VEGFR2 inhibitor, simultaneously suppresses metastasis, angiogenesis, and tumor growth. Mol Cancer Ther (2011) 10:2298–308. 10.1158/1535-7163.MCT-11-0264 21926191

[81] Smith MR Sweeney CJ Corn PG Rathkopf DE Smith DC Hussain M Cabozantinib in chemotherapy-pretreated metastatic castration-resistant prostate cancer: results of a phase II nonrandomized expansion study. J Clin Oncol (2014) 32(30):3391–9. 10.1200/JCO.2013.54.5954 25225437 PMC4383838

[82] Zhang XQ Kondrikov D Yuan TC Lin FF Hansen J Lin MF. Receptor protein tyrosine phosphatase alpha signaling is involved in androgen depletion-induced neuroendocrine differentiation of androgen-sensitive LNCaP human prostate cancer cells. Oncogene (2003) 22:6704–16. 10.1038/sj.onc.1206764 14555984

[83] Hong SK Kim JH Lin MF Park JI. The Raf/MEK/extracellular signal-regulated kinase 1/2 pathway can mediate growth inhibitory and differentiation signaling via androgen receptor downregulation in prostate cancer cells. Exp Cell Res (2011) 317:2671–82. 10.1016/j.yexcr.2011.08.008 21871886 PMC3189339

[84] Hou CH Lin FL Hou SM Liu JF. Cyr61 promotes epithelial-mesenchymal transition and tumor metastasis of osteosarcoma by Raf-1/MEK/ERK/Elk-1/TWIST-1 signaling pathway. Mol Cancer (2014) 13:236. 10.1186/1476-4598-13-236 25326651 PMC4210521

[85] Karamitopoulou E Zlobec I Gloor B Kondi-Pafiti A Lugli A Perren A. Loss of Raf-1 kinase inhibitor protein (RKIP) is strongly associated with high-grade tumor budding and correlates with an aggressive phenotype in pancreatic ductal adenocarcinoma (PDAC). J Transl Med (2013) 11:311. 10.1186/1479-5876-11-311 24330423 PMC3898115

[86] Wan XB Long ZJ Yan M Xu J Xia LP Liu L Inhibition of Aurora-A suppresses epithelial-mesenchymal transition and invasion by downregulating MAPK in nasopharyngeal carcinoma cells. Carcinogenesis (2008) 29:1930–7. 10.1093/carcin/bgn176 18667445

[87] Beltran H Rickman DS Park K Chae SS Sboner A MacDonald TY Molecular characterization of neuroendocrine prostate cancer and identification of new drug targets. Cancer Discov (2011) 1:487–95. 10.1158/2159-8290.cd-11-0130 22389870 PMC3290518

[88] D’Assoro AB Liu T Quatraro C Amato A Opyrchal M Leontovich A The mitotic kinase Aurora-A promotes distant metastases by inducing epithelial-to-mesenchymal transition in ER alpha(+) breast cancer cells. Oncogene (2014) 33:599–610. 10.1038/onc.2012.628 23334326 PMC4058768

[89] McKeithen D Graham T Chung LW Odero-Marah V. Snail transcription factor regulates neuroendocrine differentiation in LNCaP prostate cancer cells. Prostate (2010) 70:982–92. 10.1002/pros.21132 20166136 PMC2877267

